# Increased incidence of respiratory distress syndrome in neonates of mothers with abnormally invasive placentation

**DOI:** 10.1371/journal.pone.0201266

**Published:** 2018-07-26

**Authors:** Nicole T. Spillane, Stacy Zamudio, Jesus Alvarez-Perez, Tracy Andrews, Themba Nyirenda, Manuel Alvarez, Abdulla Al-Khan

**Affiliations:** 1 Department of Pediatrics, Division of Neonatology, Hackensack University Medical Center, Hackensack, New Jersey, United States of America; 2 Rutgers University, New Jersey Medical School, Newark, New Jersey, United States of America; 3 Department of Obstetrics and Gynecology, Division of Maternal-Fetal Medicine and Surgery, Center for Abnormal Placentation, Hackensack University Medical Center, Hackensack, New Jersey, United States of America; 4 Office of Research Administration, Department of Research Hackensack University Medical Center, Hackensack, New Jersey, United States of America; Federal University of Sergipe, BRAZIL

## Abstract

**Background:**

The incidence of abnormally invasive placentation (AIP) is increasing. Most of these pregnancies are delivered preterm. We sought to characterize neonatal outcomes in AIP pregnancies.

**Methods:**

In this retrospective case-control study (2006–2015), AIP neonates (n = 108) were matched to two controls each for gestational age, antenatal glucocorticoid exposure, sex, plurity, and delivery mode. Medical records were reviewed for neonatal and maternal characteristics/outcomes. Univariate and multivariate Poisson regressions were performed to determine relative risk ratios (RR).

**Results:**

There were no mortalities. All neonatal outcomes were similar except for respiratory distress syndrome (RDS), which affected 37% of AIP neonates (versus 21% of controls). AIP neonates required respiratory support (64.8% vs. 51.9%) and continuous positive airway pressure (53.7% vs. 42.1%) for a longer duration. Univariate regression yielded elevated RRs for RDS for AIP (RR 1.78, 95% CI 1.24–2.54), placenta previa (RR = 1.94, 95% CI 1.36–2.76), and placenta previa with bleeding (RR 2.29, 95% CI 1.36–3.86). One episode of bleeding had a RR of 2.43 (95% CI 1.57–3.76), 2 or more episodes had a RR of 2.95 (95% CI 1.96–4.44), and bleeding/abruption as the delivery indication had a RR of 2.57 (95% CI 1.82–3.64). A multivariate regression stratifying for AIP and evaluating the combined and individual associations of AIP, bleeding, placenta previa, and GA, resulted in elevated RRs for placenta previa alone (RR 2.16, 95% CI 1.15–4.06) and placenta previa and bleeding (RR 1.69, 95% CI 1.001–2.85).

**Conclusions:**

The increased incidence of RDS at later gestational ages in AIP is driven by placenta previa. AIP neonates required respiratory support for a longer duration than age-matched controls. Providers should be prepared to counsel expectant parents and care for affected neonates.

## Introduction

Respiratory distress syndrome is predominantly a disease of the premature infant. It is characterized by developmental insufficiency of surfactant production and function as well as pulmonary structural immaturity. The incidence is inversely proportional to gestational age (GA), occurring in >90% in neonates less than 28 weeks [1]. Until recently, antenatal corticosteroid (ANC) administration was not standard of care in late preterm infants (34^+0^–36^+6^ weeks) as the overall incidence of RDS is low [2, 3]. A recent study has demonstrated the benefit of this treatment in late preterm infants to prevent respiratory complications [4]. Subsequently, American College of Obstetrics and Gynecology (ACOG) modified its committee opinion to recommend ANC administration for anticipated late preterm deliveries [5].

However, there are concerns about the short and long-term complications of exposure to ANC [6]. An individualized assessment of risk for RDS and other respiratory complications may better inform decision-making. Several underappreciated risk factors for RDS are placenta previa [PP), a common cause of late preterm birth [7–12], and intrapartum bleeding [13–15]. The influence of other forms of abnormal placentation (placenta accreta, increta, percreta, collectively called Abnormally Invasive Placentation—AIP), have not been studied in regards to RDS and other neonatal outcomes.

The incidence of abnormally invasive placentation (AIP) has increased and has been reported as high as 1–2 in 1000 in several locations in North America [16–18]. AIP is defined by the pathologic adherence of the placenta to the myometrium without intervening decidua. Failure of the placenta to spontaneously detach from the uterus can result in massive maternal hemorrhage, multi-organ dysfunction/failure, and death. Both ACOG and Society of Maternal Fetal Medicine recommend scheduled pre-term delivery [19] for AIP. In general, 34 weeks has been recommended as the optimal delivery window [20] to avoid emergent delivery due to maternal hemorrhage while also optimizing neonatal outcomes.

The Center for Abnormal Placentation (CAP) at our institution was formed in 2006 for multidisciplinary medical care of this high-risk condition. Abnormally invasive placentation is associated with a high prevalence of PP, particularly in its more severe forms, placenta increta and percreta. In this study, we take advantage of a large cohort of patients cared for in the CAP to investigate the association of RDS and AIP.

In the mid-1980s, McShane and colleagues first recognized a relationship between RDS and PP. They hypothesized that the increased risk for RDS was driven by maternal bleeding antenatally and perinatally with resulting fetal anemia [9]. To date, there has been little investigation of this proposed mechanism. The study included a heterogeneous PP population with some patients having AIP. However, the association of AIP with RDS was not examined distinct from the presence of PP, further confounding an elucidation of etiology.

Our experience with a large population of AIP neonates suggested an increased prevalence of RDS as compared to similar neonates. This impression led to the design of this study to examine RDS and other short-term neonatal outcomes in this population. Due to the high incidence of PP in AIP, the sample naturally lends itself to the examination of the separate and combined association of PP and/or bleeding with RDS.

## Materials and methods

The research design is a retrospective case-control study with 1:2 enrollment of AIP neonates to controls. Subjects were born at a single regional level III hospital and admitted to the neonatal intensive care unit/well baby nursery between January 2006 and November 2015. A total of 108 neonates born to mothers with AIP and 216 controls were included. There were 6 and 12 pairs of twins among AIP and controls. Control twin pregnancies that met matching criteria for both twins were identified for 5 AIP pregnancies. An appropriate twin pair could not be found for the sixth twin pregnancy. Therefore, 2 control neonates from two different twin pregnancies (1 baby from each pregnancy) were selected, resulting in 102 AIP and 205 control mothers.

AIP neonates were identified and enrolled from the database maintained by CAP. To identify those not antenatally diagnosed, additional cases were identified from pathology records where the words “cesarean hysterectomy”, “accreta”, “increta”, or “percreta” were utilized. Controls were obtained from electronic birth certificate records and from labor and delivery logs. Controls were matched for fetal sex, exposure to antenatal corticosteroids, mode of delivery, gestational age (completed week), plurity, and birth closest to the time of the relevant AIP delivery. Neonates with major congenital anomalies or chromosomal abnormalities were excluded. This study was approved by the Institutional Review Board of HackensackUMC.

Most AIP cases (87%) were histopathologically confirmed. The remainder were diagnosed by clinical assessment of abnormal adherence of the placenta in the absence of hysterectomy. Classification of AIP was as follows: (a) placenta accreta (chorionic villi superficially attach to the myometrium), (b) placenta increta (chorionic villi invade into the myometrium), and (c) placenta percreta (chorionic villi invade through the myometrium and serosa). Maternal demographic data and obstetrical data were abstracted from the EMR, including details on maternal bleeding episodes. GA was assessed by last menstrual period (LMP) and confirmed using 1^st^ trimester ultrasound biometry. The indication(s) for delivery was obtained from the delivery note or operative report.

Neonatal demographics and details of neonatal intensive care unit (NICU) and well-baby nursery hospitalization were collected. The diagnosis of RDS, the primary outcome of interest, was made using the following criteria: 1) PaO_2_ <50 mmHg in room air, central cyanosis in room air, a requirement for supplemental oxygen to keep pulse oximetry saturation >85%, or surfactant administration in the first 48 hours of life; and 2) a chest x-ray with a reticulogranular appearance to the lung fields with or without low lungs volumes and air bronchograms within the first 24 hours of life [21]. The diagnosis of transient tachypnea of the newborn (TTN) was made using the following criteria: 1) respiratory distress (flaring, grunting, and/or accessory muscle use with or without oxygen saturation ≤ 95% in room air) in the first 12 hours of life and 2) chest x-ray findings of pulmonary fluid retention [22]. RDS, it should be noted, is a separate diagnosis from TTN, made by characteristic chest x-ray findings as described above and the requirement for oxygen supplementation and/or surfactant administration for RDS, but not TTN.

The duration and type of respiratory support, requirement for and doses of surfactant, and air leak syndromes were examined. Large for gestational age (LGA) was defined as >90% for GA and small for gestational age (SGA) was defined as <10% for GA using sex-specific Fenton Preterm Growth Charts [23]. Additional outcomes included admission to NICU, length of stay (LOS), discharge weight, mortality, hyperbilirubinemia requiring phototherapy, hypoglycemia requiring a bolus of intravenous dextrose, intraventricular hemorrhage (IVH), periventricular leukomalacia, central line placement, time to attain full enteral feedings (120ml/kg/day), late-onset infections, retinopathy of prematurity, hypotension requiring treatment, anemia requiring transfusion, presence of a hemodynamically significant patent ductus arteriosus, apnea of prematurity, and administration of total parenteral nutrition (TPN). The diagnosis and grading of IVH was obtained from radiologic reports.

### Statistics

Univariate and bivariate analysis of the data were performed to examine characteristics of the data; Chi-square and Kruskal-Wallis tests of association were performed to determine differences across groups where necessary. Univariate and multivariate Poisson regression analysis was performed to examine the relative risk of experiencing RDS.

The key univariate associations included GA, AIP, PP, and bleeding/abruption, associations that are correlated and appeared to change over the study duration. Therefore to estimate the effect of bleeding, PP, AIP and GA on RDS, two different multivariate regression techniques were used. First, an unconditional repeated measures Poisson regression was used to control for any factors that might cause variability across time, thereby reducing the possible error introduced into the model by changes over time. The second modeling approach was a conditional exact Poisson regression model that stratified the analysis across AIP. This model matched cases to their controls across AIP and therefore directly assessed the effect of bleeding, PP and GA on RDS. To account for possible variation in the risk of bleeding and/or PP at different gestational ages, an interaction of these variables was also modeled.

In sensitivity analyses (not shown) demographic and clinical characteristics were included as covariates. They did not significantly explain the variation in RDS outcomes or improve model fit, and so were not included in the multivariate regression. Significance was set at p < 0.05 and trends are reported where p < 0.10. Analyses were performed using SAS 9.4 (SAS Institute Inc., Cary, NC, USA).

## Results

As noted in the Methods section, 102 AIP mothers (who gave birth to 108 neonates) and 205 control mothers (who gave birth to 216 neonates) are characterized in Table 1. AIP mothers were older, and of higher gravidity and parity (Table 1). The AIP mothers were comprised of 40 accretas, 16 incretas and 46 percretas. Fifty-nine percent were diagnosed antenatally. Placenta previa affected 72% of cases but only 6% of controls (p<0.001). Hypertensive disorders were less common in AIP. The use of general anesthesia was more common in AIP mothers (Table 1), however, there were few women exposed in either group.

**Table 1:** Maternal characteristics and complications of pregnancy.

	AIP Mothers, n = 102	Control Mothers, n = 205	P value
Maternal Age^a^	35.4 ± 5.7 (21–51)	33.0 ± 5.7 (18–47)	<0.001
Gravidity^b^^,^	4 (3, 5) [1–14]	2 (1, 4) [1–11]	<0.001
Parity^b^	2 (1, 3) [0–10]	1 (0, 2) [0–7]	<0.001
Ethnicity^c^			0.33
Caucasian	44 (43.1%)	82 (40%)	
Hispanic	34 (33.3%)	64 (31.2%)	
Black	13 (12.7%)	20 (9.8%)	
Asian	11 (10.8%)	35 (17%)	
Other/Unknown	0	3 (1%)	
Pre-pregnancy BMI^b^	26 (23, 30) [17–45]	25 (23, 30) [17–49]	0.77
BMI Categories^c^			0.05
<18.5 kg/m^2^	5 (4.9%)	5 (2.7%)	
18.5–24.9 kg/m^2^	37 (36.3%)	87 (46.5%)	
25.0–29.9 kg/m^2^	36 (35.3%)	47 (25.1%)	
>30.0 kg/m^2^	24 (23.5%)	48 (25.7%)	
Twins^c^^,^ ^d^	6 (5.9%)	12 (5.9%)	
General anesthesia (vs. regional) ^c^	14 (13.7%)	8 (3.9%)	<0.005
Maternal bleeding episodes^c^			<0.001
No bleeding	76 (74.5%)	186 (91.2%)	
1 episode	13 (12.7%)	11 (5.4%)	
≥ 2 episode	13 (12.7%)	7 (3.4%)	
Bleeding as an indicator for delivery	24 (23.5%)	17 (8.3%)	<0.001
Placenta previa^c^	72 (71.6%)	12 (5.9%)	<0.001
Bleeding as an indicator for delivery	24 (23.5%)	9 (4.4%)	<0.001
Other Complications of Pregnancy^c^			
Diabetes mellitus, gestational/pregestational	9 (8.8%)	33 (16.1%)	0.11
Maternal Hypertensive Disorders	9 (8.8%)	43 (21%)	<0.005
Cholestasis of Pregnancy	0 (0%)	6 (2.9%)	0.18
Assisted Reproduction	13 (12.7%)	18 (8.8%)	0.32

^**a**^Mean ± SD (range).

^**b**^Median (interquartile range) [range].

^**c**^Number (percentage).

^**d**^Matching criteria.

BMI = body mass index

Both antenatal and peripartum vaginal bleeding were more common, more frequent and more often an indication for delivery in AIP (Table 1).

Matching resulted in neonates that were similar in the baseline characteristics of GA, antenatal corticosteroid exposure, sex ratio, mode of delivery, and plurity. Birth weight/birth weight percentile was similar in both groups (Table 2). There was a trend towards fewer SGA and more LGA neonates in the AIP group (Table 2), despite ~ half the incidence of maternal diabetic complications. AIP neonates had a lower median APGAR score at 1 minute but no other differences.

**Table 2:** Neonatal baseline characteristics.

	AIP Neonates, n = 108	Control Neonates, n = 216	P value
Gestational Age^b^^,^ ^d^	34 3/7 (33, 37) [28–40]	34 3/7 (33–37) [27–41]	0.91
Birth Weight (g)^a^	2400 ± 670 (880–4060)	2358 ± 732 (754–4185)	0.62
Birth Weight Percentile^b^	54 (35, 74) [4–97]	52 (33, 72) [1–100]	0.35
Neonatal Sex (M/F)^d^	52%/48%	52%/48%	
Birth Weight Categories^c^			0.05
Small for Gestational Age	4 (3.7%)	11 (5.1%)	
Appropriate for Gestational Age	92 (85.2%)	196 (90.7%)	
Large for Gestational Age	12 (11.1%)	9 (4.2%)	
APGAR Scores			
1 min^b^	8 (7, 9) [1–9]	8 (8, 9) [1–9]	0.02
5 min^b^	9 (8, 9) [2–10]	9 (9, 9) [2–9]	0.07
< 7 at 1 min	19.4%	12.5%	0.10
< 7 at 5 min	4.7%	3.2%	0.53
Mode of Delivery			
Vaginal^d^	3.7%	3.7%	
C-section^d^	96.3%	96.3%	
Multiples^d^	12%	12%	
Exposure to Antenatal Steroids^c^^,^ ^d^	64 (59.3%)	128 (59.3%)	
Exposure to Magnesium^c^	29 (26.8%)	59 (27.3%)	0.93
Antenatal Steroids ≥48 hrs and ≤7 Days^c^	18 (16.8%)	46 (21.4%)	0.30

^**a**^Mean ± SD (range).

^**b**^Median (interquartile range) [range].

^**c**^Number (percentage)

^d^Matching criteria.

GA = gestational age

Admission to the NICU was required for most neonates. There were no mortalities. Outcomes for nearly all prematurity-related complications were similar (Table 3). More than half the neonates required phototherapy for hyperbilirubinemia. TPN was required in 43% of AIP neonates and controls, with time to reach full enteral feeds of 6 days. Head ultrasounds were performed in 28.7% of AIP babies and 33.3% of controls. Four AIP neonates and 13 controls experienced an IVH (one grade 3–4, all others 1–2). Both groups were similar in total NICU/hospital LOS, discharge weight and corrected GA at discharge (Table 3).

**Table 3:** Neonatal outcomes.

	AIP Neonates, n = 108	Control Neonates, n = 216	P value
Mortality	0	0	
Length of Stay (days)^a^	11 (5, 22) [2–108]	12 (4, 22) [1–78]	0.43
Discharge Weight (g)^a^	2505 (2195, 2900) [1825–3805]	2468 (2210, 2830) [1775–4156]	0.77
Corrected GA at Discharge^a^	36 4/7 (35 5/7, 38 1/7) [33 6/7-44]	36 4/7 (35 4/7, 37 6/7) [32 1/7-44]	0.59
NICU Admission^b^^,^^c^	78 (72.2%)	153 (70.8%)	0.78
NICU LOS (days)^a^	18 (9, 31) [2–108]	17 (10, 26) [1–78]	0.79
Respiratory Distress Syndrome^b^^,^^c^	40 (37%)	45 (20.8%)	< 0.005
Air Leak Syndromes (pneumothorax/PIE)^b^^,^^c^	4 (3.7%)	4 (1.9%)	0.45
Transient Tachypnea of the Newborn^b^^,^^c^	32 (29.6%)	72 (33.3%)	0.50
Hyperbilirubinemia Requiring Phototherapy^b^^,^^c^	63 (58.3%)	122 (56.5%)	0.72
Days of Phototherapy^a^	2 (1, 3) [1–8]	2 (1, 3) [1–11]	0.90
Late-onset Infections^b^^,^^c^	2 (1.8%)	8 (3.7%)	0.51
Intraventricular Hemorrhage^b^^,^^c^	4 (3.7%)	13 (6.0%)	0.44
Anemia Requiring Transfusion^b^^,^^c^	4 (3.7%)	16 (7.4%)	0.23
Central Line Placement^b^^,^^c^	12 (11.1%)	24 (11.1%)	1.00
Line Days^a^	8 (6, 12) [2–25]	12 (6.5–17.5) [2–21]	0.40
Days of TPN^a^	6 (4, 8) [2–25]	5 (4, 9) [1–32]	0.86
Days to Full Feeds^a^	6 (4, 9) [2–27]	6 (4, 9) [2–27]	0.72
Hypoglycemia^b^^,^^c^	18 (16.7%)	29 (13.4%)	0.50
Apnea of Prematurity^b^^,^^c^	28 (25.9%)	55 (25.4%)	1.00
Hypotension^b^^,^^c^	4 (3.7%)	11 (5.0%)	0.78
Retinopathy of Prematurity^b^^,^^c^	1 (0.9%)	4 (1.8%)	0.67
Hemodynamically Significant Patent Ductus Arteriosus^b^^,^^c^	0 (0%)	2 (0.9%)	0.55
Periventricular Leukomalacia	0	0	

^**a**^Median (interquartile range) [range].

^**b**^Number.

^c^Percentage.

LOS = length of stay, PIE = pulmonary interstitial emphysema, TPN = total parental nutrition, full feeds = 120ml/kg/d of enteral nutrition

**Fig 1:**
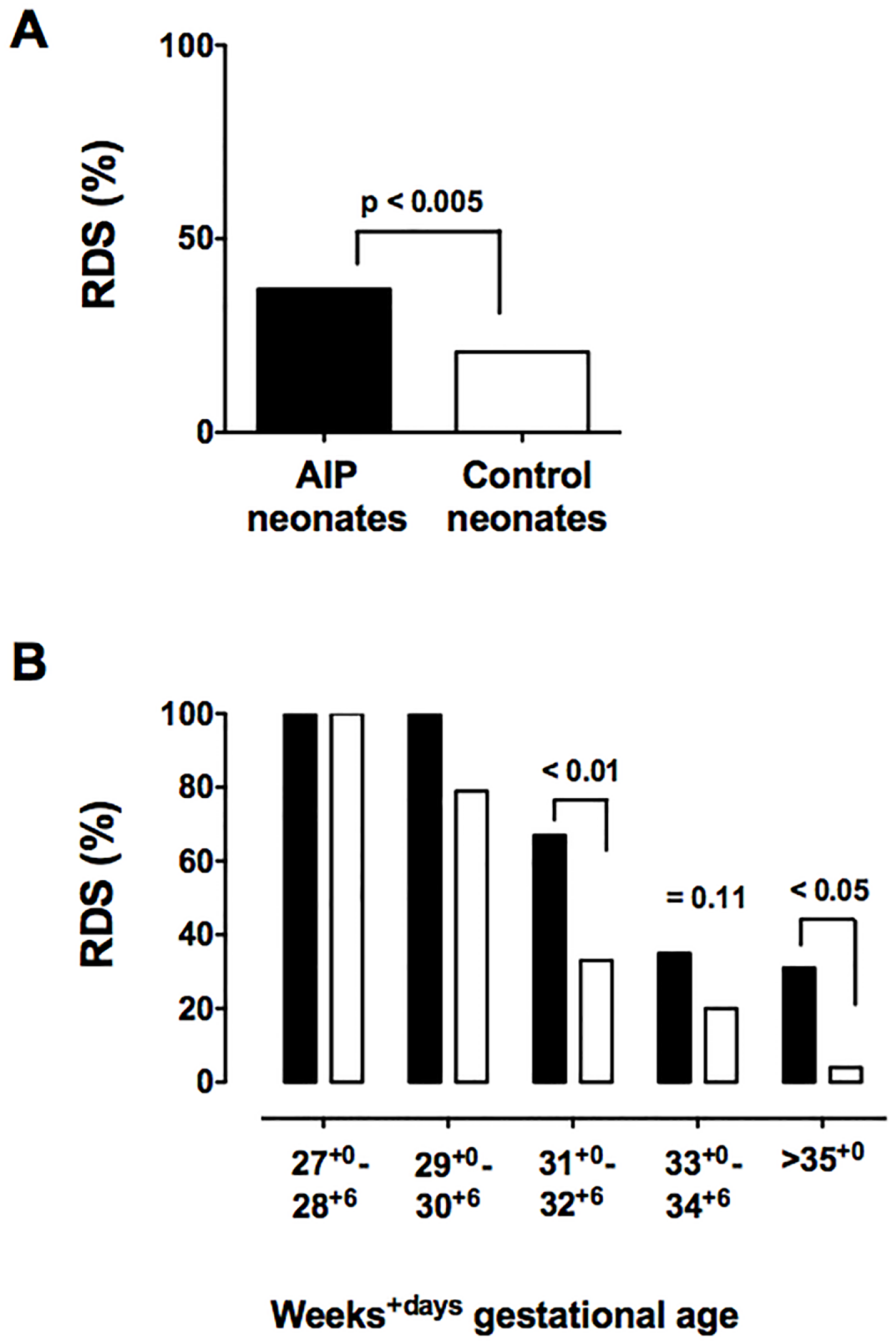
Incidence of respiratory distress syndrome. A. The incidence of RDS was 37.0% in AIP neonates (black bars) and 20.8% in control neonates (white bars). B. Rates of RDS tended to be or were significantly greater in the later gestational age categories, beginning at 31^+0^ weeks. RDS incidence in AIP neontates was 34% higher at 31+0–32^+6^ wks, 15% at 33+0–34^+6^ wk and 27% at 35+0–36^+6^ wks.

There was nearly twice the incidence of RDS in AIP neonates than controls, affecting 37% vs. 21% (p<0.005, Fig 1). The greater incidence of RDS in AIP babies occurred at later GA (Fig 1). Within GA categories, the RR for RDS was significantly greater for AIP neonates at 31^+0^ to 32^+6^ weeks (RR 2.91, 95% CI 1.29–6.58, p = 0.009) and at >35 weeks (RR 3.02, 95% CI 1.48–6.12, p = 0.03), and tended to be greater at 33^+0^ to 34^+6^ (RR 1.61, 95% CI 0.95–2.70, p = 0.11).

The use of antenatal corticosteroids increased progressively during the study, from 43% in 2006 to 83% in 2015, in parallel with improved antenatal detection of AIP [24]. Despite the increased use of corticosteroids, the incidence of RDS by year of delivery remained similar.

**Fig 2:**
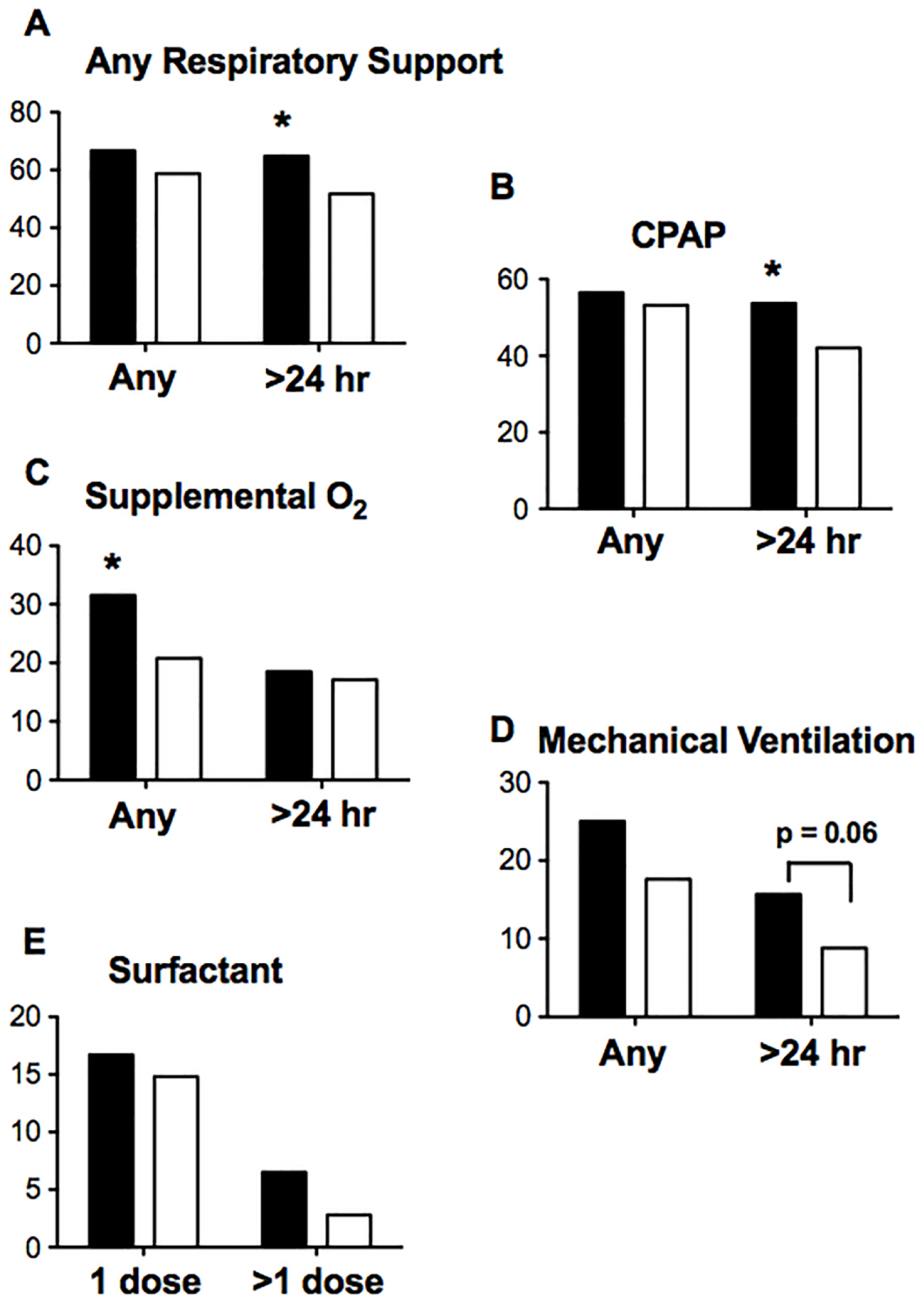
Respiratory support in AIP neonates as compared with controls. AIP neonates (black bars). Controls (white bars): A. The need for any type of respiratory support >24 hours was greater in AIP neonates. B. CPAP for >24 hours was required more frequently in AIP neonates. C. More AIP neonates required oxygen. D. The requirement for mechanical ventilation was similar. E. Surfactant administration was similar.

AIP neonates were more likely to require any type of respiratory support (Fig 2A) and continuous positive airway pressure (CPAP) alone for > 24 hours (Fig 2B). They were also more likely to require supplemental oxygen (Fig 2C). There was a trend for AIP neonates to require mechanical ventilation for >24 hours (p = 0.06, Fig 2D). Surfactant administration (Fig 2E), need for high frequency oscillatory ventilation, and incidences of air leak syndromes and TTN was similar between groups (Table 3).

In univariate analyses of relative risk ratios, any form of AIP was associated with an elevated RR for RDS (1.78, 95% CI 1.24–2.54, p<0.005). With respect to the severity of AIP, the RR for accreta was 1.46 (95% CI 0.88–2.43, p = 0.14), for increta was 1.80 (95% CI 0.91–3.57, p = 0.09) and for percreta was RR 2.09 (95% CI 1.37–3.18, p<0.001). The RR for RDS associated with PP was 1.94 (95% CI 1.36–2.76, p<0.001), which was greater than for all AIP, but less than percreta (Table 4). When PP occurred with vaginal bleeding, the RR rose to 2.29 (Table 4). Vaginal bleeding for any cause during pregnancy was associated with an elevated RR for RDS of 2.43 (95% CI 1.57–3.76, p<0.001) for a single episode of bleeding and increased to 2.95 (95% CI, 1.96–4.44, p<0.001) for 2 or more episodes. If the delivery indication was bleeding/suspected abruption, the RR was 2.57 (95% CI 1.82–3.64, p<0.001).

**Table 4:** Univariate analysis for relative risk ratios for respiratory distress syndrome.

Variable	Relative Risk Ratio (95% CI)	P value
Birth Weight Categories		
SGA <10^th^ percentile	0.51 (0.14–1.89)	0.31
AGA 10^th^– 90^th^ percentile (ref. category)	1.00	
LGA >90^th^ percentile	1.46 (0.82–2.61)	0.20
Race		
Caucasian (ref. category)	1.00	
Hispanic	0.76 (0.48–1.19)	0.23
Black	1.50 (0.93–2.42)	0.10
Asian	0.68 (0.36–1.29)	0.24
Other or unknown	1.18 (0.23–5.97)	0.84
BMI (pre-pregnancy) Categories		
BMI <18.5 kg/m^2^	1.29 (0.66–2.52)	0.45
BMI: 18.5–24.9 kg/m^2^ (Ref. category)	1.00	
BMI: 25.0–29.9 kg/m^2^	1.34 (0.85–2.10)	0.21
BMI: 30.0–34.9 kg/m^2^	1.24 (0.68–2.26)	0.48
BMI >35.0 kg/m^2^	1.34 (0.74–2.42)	0.33
Mode of Delivery		
Vaginal birth (Ref. category)	1.00	
Caesarean section	3.23 (0.49–21.29)	0.22
Plurality of Birth		
Singleton (Ref. category)	1.00	
Twins	1.01 (0.56–1.83)	0.97
Neonatal Sex		
Female (Ref. category)	1.00	
Male	1.15 (0.80–1.66)	0.47
Maternal Hypertensive Disorder		
No (Ref. category)	1.00	
Yes	0.88 (0.53–1.48)	0.64
Maternal Diabetes		
No (Ref. category)	1.00	
Yes	0.99 (0.59–1.68)	0.98
Abnormally Invasive Placenta		
No (ref. category)	1.000	
Yes	1.78 (1.24–2.54)	<0.005
AIP Diagnosis		
No AIP (Ref. category)	1.000	
Accreta	1.46 (0.88–2.43)	0.14
Increta	1.80 (0.91–3.57)	0.09
Percreta	2.09 (1.37–3.18)	<0.001
Placenta Previa		
No (Ref. category)	1.00	
Yes	1.94 (1.36–2.76)	<0.001
No bleeding	1.00	
With bleeding episodes	2.29 (1.36–3.86)	<0.005
Bleeding Episodes in Pregnancy		
0	1.000	
1	2.43 (1.57–3.76)	<0.001
≥2	2.95 (1.96–4.44)	<0.001
Bleeding/Abruption as Delivery Indication		
No (Ref. category)	1.000	
Yes	2.57 (1.82–3.64)	<0.001
In-vitro Fertilization		
No (Ref. category)	1.000	
Yes	1.01 (0.62–1.65)	0.97

In the course of the analyses it became clear that the twin pregnancies differed from the singleton pregnancies. The twin pregnancies all had accreta, rather than more severe AIP, none had PP with bleeding, and all were from the later years of the study (≥ 2010). Moreover, 8/36 twins had RDS, all of which occurred at or after 33 weeks of gestation, representing 30% of cases in that GA group (S1 Table). Given these differences, we performed the multivariate regression analyses with and without the twins.

With all neonates included in the unconditional repeated measures multivariate Poisson regression, AIP is not associated with RDS, rather RDS is associated with gestational age and PP. Each additional week of gestation beyond 28 wks is associated with a 21% reduction in the risk of RDS (Table 5, RR 0.79, 95% CI 0.73–0.85, p<0.0001). Placenta previa is associated with a 72% increased risk of RDS compared to neither bleeding nor PP (Table 5, RR 1.72, 95% CI 1.17–2.54, p<0.01). Neither bleeding alone nor the combination of bleeding and PP is correlated with RDS in this model. However, when twins are excluded, both PP alone and PP with bleeding are significant (Table 5). The association of RDS with PP and PP with bleeding is stronger with the twin pregnancies omitted (Table 6) and more consistent with the conditional models. AIP is not significantly associated with RDS in either model.

**Table 5:** Multivariate analysis with unconditional repeated measures Poisson regression with correction for variation across time for relative risk ratios for respiratory distress syndrome.

All Neonates			
Covariate	Relative Risk Ratio	95% CI	P value
AIP	1.07	0.70–1.63	0.76
Gestational age (wks)	0.79	0.73–0.85	<0.0001
Bleeding and/or Previa Reference = neither			
Bleeding alone	0.96	0.53–1.73	0.88
Previa alone	1.72	1.17–2.54	0.006
Bleeding and Previa	1.05	0.76–1.45	0.75
**Twins Excluded**			
AIP	1.27	0.87–1.86	0.21
Gestational age (wks)	0.70	0.67–0.74	<0.0001
Bleeding and/or Previa Reference = neither			
Bleeding alone	1.18	0.70–2.01	0.5
Previa alone	2.06	1.34–3.16	0.001
Bleeding and Previa	1.59	1.00–2.54	0.05

**Table 6:** Multivariate analysis with conditional exact Poisson regression with stratification across AIP for relative risk ratios for respiratory distress syndrome.

All Neonates			
Covariate	Relative Risk Ratio	95% CI	P value
Gestational age (wks)	0.71	0.66–0.77	<0.0001
Bleeding and/or Previa Reference = neither			
Bleeding alone	1.05	0.38–2.94	0.92
Previa alone	2.16	1.15–4.06	0.02
Bleeding and Previa	1.69	1.001–2.85	<0.05
**Twins Excluded**			
Gestational age (wks)	0.70	0.64–0.76	<0.0001
Bleeding and/or Previa Reference = neither			
Bleeding alone	1.14	0.40–3.20	0.87
Previa alone	2.53	1.31–4.90	0.006
Bleeding and Previa	1.84	1.07–3.15	0.027

In the exact conditional Poisson model, which stratifies outcomes across AIP, both PP alone and the combination of bleeding and PP significantly contribute to RDS risk (Table 6). In this model, the risk of RDS associated with significant covariates is slightly higher than observed in the previous model (Table 5) but, again, AIP is not driving variation in RDS outcomes. The stratified design of this model matches cases with their specific controls to enable examination of RDS outcomes more directly.

**Fig 3:**
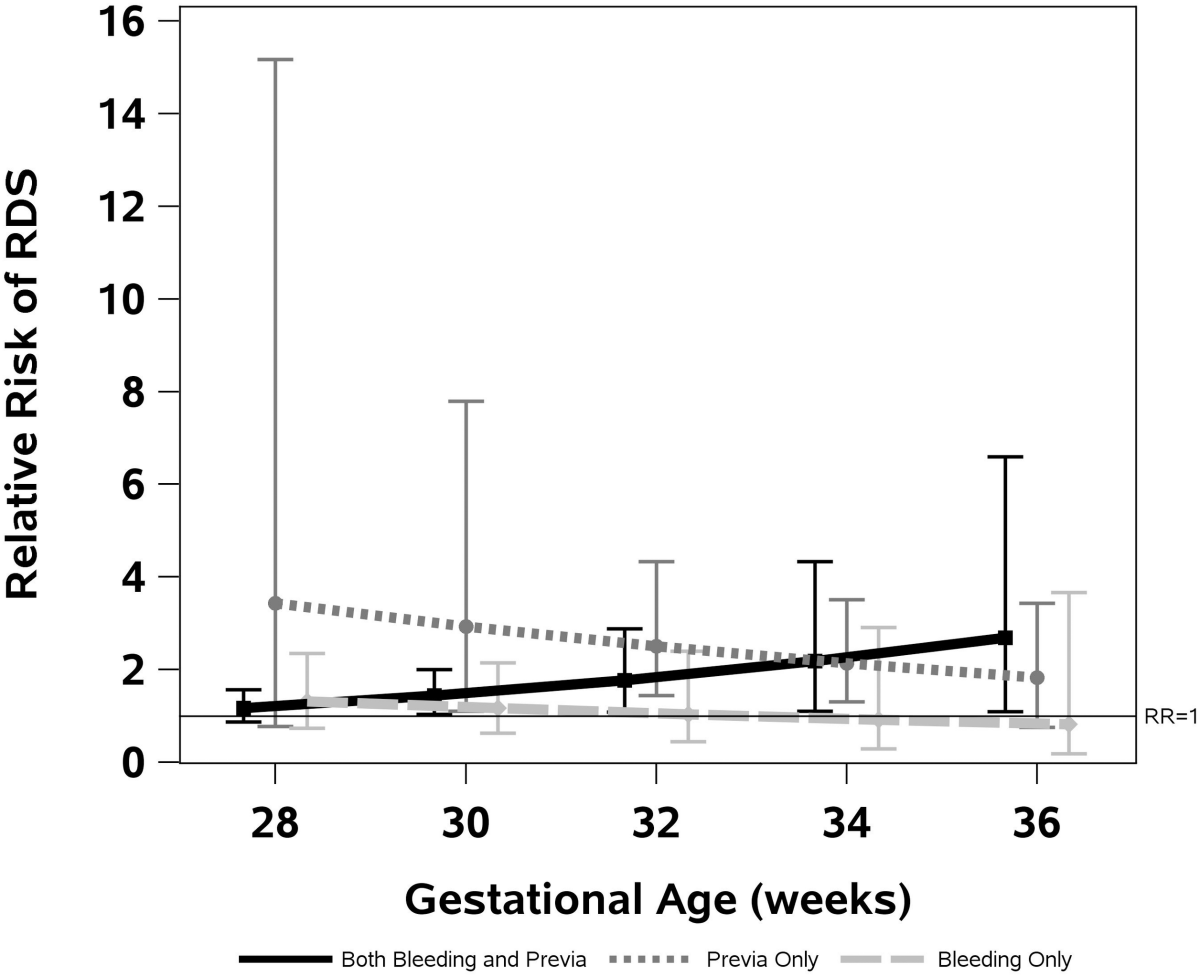
Relative risk for respiratory distress syndrome at different gestational ages with placenta previa, bleeding, and placenta previa with bleeding. This graphic representation of the data contained in multivariate analysis 5B indicates the risk of RDS declines with gestational age for singleton pregnancies without any PP or bleeding. Placenta previa alone or bleeding alone are associated with higher risk of RDS earlier in gestation, falling to parity with the ‘neither’ group as GA increases. In contrast, risk rises late in pregnancy for PP with bleeding.

Fig 3 summarizes the results of a model without the twin pregnancies that includes an interaction between GA and PP and bleeding. The figure highlights the increased later gestational age-risk for singleton pregnancies exposed to PP and bleeding. In contrast, the RR for RDS diminishes with increasing gestational age in neonates of pregnancies complicated by bleeding alone, PP alone, or neither.

## Discussion

This study is the first to comprehensively document neonatal outcomes in histopathologically confirmed AIP pregnancies. We describe an increased incidence of RDS, especially in late preterm infants in this population, which is of clinical relevance to the newer ACOG guidelines supporting ANC for late preterm delivery [25]. It is the also the first to consider the separate and combined association of AIP, PP, antenatal and peripartum bleeding with RDS. We conclude that it is not AIP driving the association with RDS, rather it is the correlated condition of PP with or without bleeding.

RDS occurred in more than a third of AIP neonates and approached double the incidence of control infants. The difference is more pronounced at GA ≥31 weeks, when most neonates experience a notable decline in RDS risk [26], and correlated with a longer duration of respiratory support and increased oxygen administration. The inclusion of twin pregnancies confounds these findings as they account for a substantial portion of the late GA RDS cases. Twins require study as a separate population, as it is likely their risk of RDS over gestational age differs from that of singleton pregnancies and the modifying effect of bleeding and/or previa could not be assessed in the present study.

Prior reports of neonatal outcomes in AIP pregnancies have been limited by small sample size, heterogeneous study populations or inclusion of predominantly clinically diagnosed cases of AIP [27] [28]. The outcomes reported are general, such as NICU admission, LOS, APGAR scores, incidence of prematurity and low birth weight [29] [30] [28] [31]. Specific outcomes relevant to prematurity such as RDS, TTN, IVH, etc. have not previously been assessed. The most comparable study to ours, [31] focused on antenatally diagnosed versus undiagnosed cases. Antenatally diagnosed cases had higher NICU admission rates (86% vs. 60%) and longer hospital length of stay (10.7 d vs. 6.9 d, p = 0.006), both due to earlier delivery of the diagnosed cases. An Israeli case-control study [28] examined 310 cases of AIP. However, AIP diagnosis was predominantly clinical, most babies were delivered at term, and only 2% had PP. The lower birth weights reported were likely due to a greater incidence of preterm birth (10.7% vs. 1%) and SGA in AIP cases (27.3% vs. 14%). However, considering the unusually high incidence of SGA in both cases and controls, and near-absence of PP, both the findings and diagnosis of AIP are of questionable validity. We reviewed neonatal outcomes before and after initiation of the team-management of AIP now followed at our Institution, which includes targeted delivery at 34 weeks [16]. However, greater than 30% of our mothers were delivered earlier than intended due to bleeding. The management protocol conferred significant maternal benefits, but neonatal morbidity remained the same. Kassem [27] compared outcomes in PP alone with PP and AIP, histopathologically confirmed in 76% of 25 AIP cases. There was no difference in GA, birth weight, SGA, or APGAR score <7 at 1 or 5 minutes.

Other than RDS, our comprehensive study of both common and uncommon neonatal outcomes did not differ. SGA was not more frequent in AIP infants, in contrast with the Israeli report [28]. In fact, there was trend towards more LGA infants, only one of which was IDM. Nonetheless, the consequences of prematurity are significant, with 72% of AIP neonates admitted to the NICU, a median LOS of 18 days, and 67% requiring some form of respiratory support. Other frequent complications are directly related to prematurity and did not differ between groups, including apnea of prematurity, central line placement, delay in attaining full enteral feedings, hypoglycemia, and hyperbilirubinemia.

The need for NICU admission is a substantial parental stressor [32] [33]. Moreover, all preterm infants are at increased risk for adverse outcomes such as mental retardation, cerebral palsy, and disorders of psychological development, behavior, and emotion. Most preterm infants with special needs are born moderately (31^+0^ to 33^+6^ weeks) or late preterm (34^+0^ to 36^+6^ weeks) [34]. RDS generates additional risk for bronchopulmonary dysplasia with concomitant risk for neurodevelopmental impairment and for recurrent wheezing illnesses [35] [36].

The increased incidence of RDS has not been reported in the neonates of AIP pregnancies, but it has been described in the infants of mothers with PP [7–12, 37] and vaginal bleeding due to other causes [8, 13–15]. In our study, PP complicated 72% of AIP pregnancies and 6% of the control pregnancies. McShane and others [9] [12] hypothesized that fetal hypoxia/anemia and placental fibrosis due to maternal bleeding episodes predisposed infants to RDS. Supporting the association of bleeding with RDS are other studies in which abruption or idiopathic antenatal bleeding increase the risk of RDS [8, 13–15]. Since both placental abruption and PP cause bleeding, as may AIP even in the absence of PP, bleeding appears to be the common factor associated with RDS. We undertook multivariate analyses in an effort to determine whether it is bleeding, PP, AIP or an interaction between these conditions that comprise the link to RDS, hitherto absent in the literature.

Using two different regression analyses allowed us to assess both the effect of twins and the effect of AIP on RDS outcomes. In the first analysis, the repeated measure design, the inclusion of the twins had the effect of muting the relationship between RDS and bleeding and PP. When analyzing singletons only, we found results similar to those produced by the conditional regression analysis. Using the two approaches together, we were able to determine that AIP was not a significant factor in RDS outcomes. For singleton pregnancies our results showed a consistent effect, with elevated risk of RDS for both PP alone, and bleeding and PP combined, regardless of the regression approach. When looking at the data with twins, this effect is not as clear. The difference in RRs in Table 6 and summary of characteristics in S1 Table are illustrative of the problem. Twin pregnancies predominantly have neither placenta previa nor bleeding. Larger samples of women with antenatal vaginal bleeding during pregnancy will be needed to further refine the relative risks associated with placental location versus bleeding. Likewise, a separate study of a larger sample of twins is needed, as twins are already at higher risk for RDS, but the risk-modification with PP and/or bleeding is likely to differ from singletons. Regardless, it is clear that AIP is not a direct risk for RDS.

This study was limited by several factors. Our AIP population may not reflect those at other centers. As a referral center, we have a disproportionately high incidence of severe disease and smaller numbers of accreta and increta may have led to underestimation of RRs associated with these milder forms of AIP. We could not match for all known risk factors for RDS, nor could we evaluate all potential confounders, e.g. presence of labor prior to cesarean. We found no association of RDS with diabetic or hypertensive complications of pregnancy, anesthesia, or birth weight category, but this may be due to sample size. Our study population was acquired over 9 years. During this time, antenatal diagnosis and active management of AIP with administration of antenatal corticosteroids increased, and innovations in neonatal care occurred. It is possible that changes in theory and/or practice over the duration of the study period may have unmeasured or unobserved effects on the outcome. Statistical methods were utilized to reduce the influence of potential temporal effects, but they cannot completely account for them. The study was also limited by its retrospective design, and, as noted above, inclusion of twins complicated our analyses.

In summary, we characterized neonatal outcomes in AIP pregnancies and described an excess incidence of RDS, particularly at older GA. Corresponding with this increased incidence of RDS was an increased need for respiratory support and oxygen administration. We conclude that PP and bleeding drive the association of AIP with RDS. Their relative contribution to RDS risk remains to be elucidated, especially with respect to gestational age. Our work suggests a split association in which PP without bleeding was associated with increased risk of RDS at earlier gestational ages and PP with bleeding was associated with greater risk at the later gestational ages. These associations have not been previously described, nor has the etiology of PP and/or bleeding relative to RDS risk been adequately investigated. Further investigation of the role of proposed causal factors such as hypoxia, anemia, and the hypothalamic pituitary axis are needed.

The study time period was prior to the recent ACOG recommendations regarding administration of ANC in pregnancies at risk for late preterm delivery [25]. Most infants born to mothers with AIP will be premature. For AIP pregnancies, planned delivery is recommended at 34–35 weeks [19] [20] [16] and emergent delivery is required in >90% of cases expectantly managed ≥ 35 weeks [38]. Thus the newly revised ACOG recommendation [5] for ANC administration in pregnancies at risk for late pre-term delivery may particularly benefit AIP neonates and non AIP pregnancies with PP and/or bleeding. Further studies to identify the benefit of ANC and other possible mitigating factors need to be conducted. Obstetrical and neonatal medical providers should counsel expectant parents about the elevated risk for RDS in the presence of PP and/or antenatal bleeding and be prepared to care for infants with RDS, even at later GAs, where these conditions are present in mothers.

## Supporting information

S1 Table Incidence of respiratory distress syndrome by gestational age, placenta previa, bleeding, both placenta previa and bleeding, and neither in singleton and all neonates. (DOCX)
