# Supplementary material for: Increased incidence of respiratory distress syndrome in neonates of mothers with abnormally invasive placentation
Source: PLoS One. 2018 Jul 26;13(7):e0201266. doi: 10.1371/journal.pone.0201266 (PMC6062082; doi:10.1371/journal.pone.0201266)
Supplement: S1 Table — (DOCX) [file pone.0201266.s001.docx]

**Supplementary Table 1**. Incidence of Respiratory Distress Syndrome by Gestational Age, Placenta Previa, Bleeding, Both Placenta Previa and Bleeding, and Neither in Singleton and All Neonates

| Distribution of Bleeding/Abruption-Placenta Previa by Gestational Age- Mothers of Singletons Only | | | | | | | | |
| --- | --- | --- | --- | --- | --- | --- | --- | --- |
| Gestational Age | Bleeding Only | | Previa Only | | Both Bleeding and Previa | | No Bleeding or Previa | |
|  | All Patients | % RDS | All Patients | % RDS | All Patients | % RDS | All Patients | % RDS |
| 27-28^+6^ weeks | 1 (10.0) | 1 (100.0) | 0 (0.0) | 0 (0.0) | 1 (2.9) | 1 (100.0) | 10 (5.2) | 10 (100.0) |
| 29-30^+6^ weeks | 2 (20.0) | 2 (100.0) | 0 (0.0) | 0 (0.0) | 10 (28.6) | 10 (100.0) | 9 (4.6) | 6 (66.7) |
| 31-32^+6^ weeks | 3 (30.0) | 1 (33.3) | 6 (11.8) | 3 (50.0) | 8 (22.9) | 6 (75.0) | 31 (16.0) | 11 (35.5) |
| 33-34^+6^ weeks | 2 (20.0) | 0 (0.0) | 21 (41.2) | 8 (38.1) | 10 (28.6) | 3 (3.0) | 60 (31.0) | 9 (15.1) |
| 35+ weeks | 2 (20.0) | 0 (0.0) | 22 (47.1) | 3 (13.6) | 6 (17.1) | 1 (16.7) | 84 (43.3) | 1 (1.2) |
|  |  |  |  |  |  |  |  |  |
| Distribution of Bleeding/Abruption-Placenta Previa by Gestational Age- All Mothers | | | | | | | | |
| Gestational Age | Bleeding Only | | Previa Only | | Both Bleeding and Previa | | No Bleeding or Previa | |
|  | All Patients | % RDS | All Patients | % RDS | All Patients | % RDS | All Patients | % RDS |
| 27-28^+6^ weeks | 1 (10.0) | 1 (100.0) | 0 (0.0) | 0 (0.0) | 1 (2.9) | 1 (100.0) | 10 (5.2) | 10 (100.0) |
| 29-30^+6^ weeks | 2 (20.0) | 2 (100.0) | 0 (0.0) | 0 (0.0) | 10 (28.6) | 10 (100.0) | 9 (4.6) | 6 (66.7) |
| 31-32^+6^ weeks | 3 (30.0) | 1 (33.3) | 6 (12.2) | 3 (50.0) | 8 (22.9) | 6 (75.0) | 37 (16.2) | 12 (32.4) |
| 33-34^+6^ weeks | 2 (20.0) | 0 (0.0) | 21 (42.9) | 8 (38.1) | 10 (28.6) | 3 (30.0) | 78 (34.2) | 17 (21.8) |
| 35+ weeks | 2 (20.0) | 0 (0.0) | 24 (44.9) | 3 (12.5) | 6 (17.1) | 1 (16.7) | 94 (41.2) | 1 (1.1) |
